# C-Peptide Effects on Renal Physiology and Diabetes

**DOI:** 10.1155/2008/281536

**Published:** 2008-05-27

**Authors:** L. Rebsomen, A. Khammar, D. Raccah, M. Tsimaratos

**Affiliations:** UPRES EA 21-93, Laboratoire de Diabétologie, Faculté de Médecine de Marseille, Université de la Méditerranée, 13385 Marseille Cedex 05, France

## Abstract

The C-peptide of proinsulin is important for the biosynthesis of insulin and has for a long time been considered to be biologically inert. Animal studies have shown that some of the renal effects of the C-peptide may in part be explained by its ability to stimulate the Na,K-ATPase activity. Precisely, the C-peptide reduces diabetes-induced glomerular hyperfiltration both in animals and humans, therefore, resulting in regression of fibrosis. The tubular function is also concerned as diabetic animals supplemented with C-peptide exhibit better renal function resulting in reduced urinary sodium waste and protein excretion together with the reduction of the diabetes-induced glomerular hyperfiltration. The tubular effectors of C-peptide were considered to be tubule transporters, but recent studies have shown that biochemical pathways involving cellular kinases and inflammatory pathways may also be important. The matter theory concerning the C-peptide effects is a metabolic one involving the effects of the C-peptide on lipidic metabolic status.This review concentrates on the most convincing data which indicate that the C-peptide is a biologically active hormone for renal physiology.

## 1. INTRODUCTION

Diabetes mellitus is a chronic metabolic disorder relative to insulin deficit that induces metabolic and degenerative complications in various organs, including nerves, heart, and kidneys [1]. C-peptide, secreted in blood stream in equimolar amounts with insulin, was considered to be a reliable marker of residual beta-cell function [2–4]. During the past decade, numerous studies in both humans and animals have demonstrated that C-peptide, although not influencing blood sugar control, might play a role in preventing and potentially reversing some of the chronic complications of type 1 diabetes, especially diabetic nephropathy [5–23]. Thus C-peptide may be an active peptide with relevant physiologic effects different from and complementary to those of insulin [24]. Several theories have been raised to explain C-peptide effects on renal function during diabetes. This review focuses on the most convincing theories that are implicated in C-peptide effects on renal function.

## 2. C-PEPTIDE AND GLOMERULAR FUNCTION

### 2.1. C-peptide Effects on Renal Function have been Demonstrated in Humans

Johansson et al. conducted a double blind study that measured glomerular filtration rate and proteinuria in type 1 diabetic patients with basal microalbuminuria, a marker of early stages of diabetic nephropathy. After three months of substitution by both C-peptide and insulin, patients exhibited a better glycemic control, a reduction of diabetes-induced glomerular hyperfiltration and microalbuminuria [7]. In another study, they confirmed that proteinuria was significantly reduced in the C-peptide group during the cross-over period [9]. Both studies were conducted in diabetic patients so that the effects of C-peptide could not be differentiated from an improved glycemic control. However, the same group examined the short-term effect of C-peptide infusion on renal function in insulin-treated diabetic patients and found similar results on glomerular filtration rate [5]. Altogether, these papers strongly argue for a specific C-peptide-related effect on glomerular function.

Recently, Fiorina et al. observed that renal and pancreatic transplantation of diabetic patients resulted in a better renal graft outcome.

They observed kidney and erythrocyte Na,K-ATPase activity, and stabilization of microalbuminuria correlated with residual pancreatic secretion [14, 15]. In chronic renal failure type 1 diabetic patients, residual pancreatic activity, restored by kidney-pancreas transplantation resulted in enhanced kidney graft survival, hypertrophy, and vascular function [25]. Furthermore, combined kidney-pancreas transplantation was associated with better high-energy phosphates metabolism than in kidney alone transplantation, suggesting that restoration of beta-cell function positively affects kidney graft metabolism [26]. All these data raised the question of C-peptide being a “kidney protector” [27].

### 2.2. Similar Studies were Conducted in Animal Models

In experimental conditions, several groups have shown that streptozotocin-induced diabetic rats treated with C-peptide exhibit improvement of metabolic status, renal function, and reversal of some of the morphologic changes associated with diabetic nephropathy [20, 21, 28].

Sjoquist et al. showed that perfusion of C-peptide results in significantly reduction of both proteinuria and diabetes-induced glomerular hyperfiltration which is considered to be the initial state of diabetic nephropathy in streptozotocin rats. They also showed that C-peptide also restores renal functional reserve [20]. In another study, they confirmed that 14 days of C-peptide perfusion prevented glomerular hypertrophy [21].

Theses results were confirmed by Kamikawa et al. that could demonstrate, in the same experimental model, that C-peptide suppresses diabetes-induced abnormal glomerular eNOS expression [29].

The mechanisms involved in glomerular effects was supposed to be an constriction of the glomerular afferent arteriols [30].

Altogether, these studies suggest that the absence of C-peptide contributes to the initial state of diabetic nephropathy. Evidence comes from specific studies that the C-terminal EVARQ fragment of the C-peptide molecule is responsible for most of the observed effects [31].

Although seducing, the glomerulocentric view of renal function during diabetes has been challenged by Thomson et al. that explored to tubuloglomerular feedback in this situation [32]. The results of their studies clearly established that a tubulocentric view of diabetes-associated nephropathy could be considered [33].

Interestingly, renal C-peptide effects were also constant with this hypothesis.

## 3. C-PEPTIDE AND TUBULAR FUNCTION

More than twenty years ago, Wald et al. suggested that tubular Na,K-ATPase activity was related to glomerular filtration rate during diabetes [34]. Several groups have confirmed the time-dependant evolution of Na,K-ATPase activity during diabetes, and the role of Na transport (for review see [35]). Recently, Kim et al. have raised the hypothesis that uncontrolled diabetes results in increased levels of several proteins including sodium transporters such as BSC1. They suggested that the enhanced expression of sodium transporters are compensatory changes that prevent a progressive decline in urinary concentrating ability despite the continuing osmotic diuresis [36]. Thus they showed that the regulation of BSC1 was not dependant on vasopressin during streptozotocin-induced diabetes in brattelboro rats [36]. Bardoux et al. have shown that vasopressin plays a crucial role in the onset and aggravation of the renal complications of diabetes. The mechanisms involve the tubular fluid in the loop of Henle, inhibition of the tubuloglomerular feedback control of glomerular function, and alterations in glomerular hemodynamics [37].

All these data support the hypothesis that the renal tubule is involved early in the course of diabetic nephropathy. Therefore, the effects of C-peptide substitution on renal tubule during type 1 diabetes were considered.

### 3.1. The Transport Theory

The Na,K-ATPase is an ubiquitous membrane-bound enzyme complex that plays fundamental role in cellular function. The basic function of the Na,K-ATPase is to maintain the high Na+ and K+ gradient across the plasma membrane of animal cells, at the expense of ATP hydrolysis (for review see [38]). Cellular C-peptide action is mediated through Na,K-ATPase activation in various organs including kidney [18, 19, 39]. Interestingly, Na+,K+-ATPase activity is increased during the first weeks after diabetes onset, and then decreased in various organs damaged by long-term diabetic degenerative complications, including kidney [40–47].

We have shown that C-peptide restores both glomerular and tubular function in diabetic rats. In vivo, C-peptide supplementation for one month improved body weight in streptozotocin-induced diabetic rats and decreased urinary sodium wasting [28]. A compensatory mechanism to conserve water and solute may involve changes in the abundance of the medullary transport proteins involved in the sodium handling. In a recent study, we observed that in vivo C-peptide supplementation for one month induced no changes in kidney abundance and transcription status of several tubular sodium transporters including the epithelial sodium channel (EnaC), and NKCC2/BSC1 cotransporter in diabetic rats. In this study, rats were made diabetic after streptozotocin injection and then were submitted to infusion with physiological doses of either insulin, homologous C-peptide, or both (unpublished data). Thus the transport theory of C-peptide's action is probably not relying on changes in amounts of renal tubule Na transporters.

### 3.2. The Biochemical Theory

Even if the trigger of sodium Na transport is not dependent on changes in Na transporters, the effects on Na,K-ATPase activity and expression are permanent findings. Several groups have shown that C-peptide action was probably secondary to biochemical changes. The first argument was found in renal cells that exhibited intracellular calcium increase secondary to C-peptide exposure [18]. Then several groups showed that incubation of various cell line types with C-peptide resulted in PKC, MAPK, ERK activation (for review see [24]).

Other studies have suggested that diabetes is a state of increased renal nitric oxide (NO) activity as assessed by urinary excretion of nitrites and nitrates (NOx), and that NO synthase inhibitors reverse the increased glomerular filtration rate (GFR) observed in experimental diabetes. Interestingly, in contrast to the effects on renal haemodynamics, NO does not play an important role in the altered renal sodium handling observed in experimental diabetes [48].

### 3.3. The Inflammatory Theory

C-peptide causes multiple molecular and physiological effects, and improves renal and neuronal dysfunction in patients with diabetes. However, whether C-peptide controls the inhibitor kappaB (IkappaB)/NF-kappaB-dependent transcription of genes, including inflammatory genes was unknown. Peroxisome proliferator-activated receptor gamma (PPARgamma) has key roles in the regulation of adipogenesis, inflammation, and lipid and glucose metabolism. These issues were progressively answered. In an in vitro model, both insulin and C-peptide induced a concentration-dependent stimulation of PPARgamma transcriptional activity [49]. Another step was made by Kitazawa et al. that showed that C-peptide stimulates the transcription of inflammatory genes via activation of a PKC/IkappaB/NF-kappaB signaling pathway [50]. The inflammatory theory was further supported by Maezawa et al. that reported C-peptide TGF-beta surppession in STZ-model [51].

**Figure 1:**
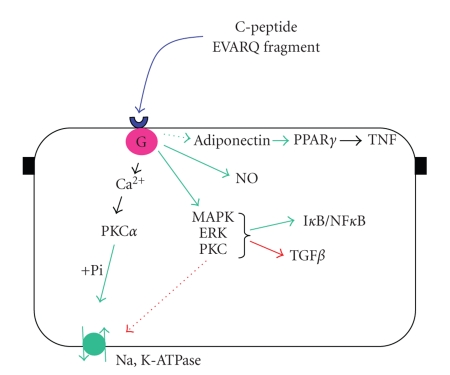
Modelization of C-peptide effects on renal tubule. The EVARQ fragment of C-peptide binds to a membrane G-protein couple receptor of a tubule cell. Intracellular calcium increase results in activation of PKCa and affects Na,K-ATPase activity. Together with this effect, an increase of intracellular kinases results in either activation or inhibition of inflammatory mediators. Green indicates stimulation, red inhibition. Arrows in dash lines are suggested pathways.

These experimental data confirm that C-peptide exerts a wide range of cellular effects (see Figure 1). Some of them may be relevant to explain renal tubular effects.

### 3.4. The metabolic theory

The effects of C-peptide on lipidic metabolic status have not been documented yet. Thus the effects of C-peptide on PPAR expression triggered the curiosity on the role of the adipocyte network and its relationship with renal physiology. Preliminary results of our group showed that C-peptide infusion for one month improved lipidic status of streptozotocin rats, by reducing cholesterol and triglycerid levels, but did not influence renal PPAR gamma expression (rebsomen et al. work in progress). On the other side, C-peptide reduced the adiponectin released by human adipocytes (Khammar et al. work in progress). Thus if the mechanism of C-peptide effect on lipid metabolism remains to be elucidated, it is known that diabetes results in inhibition of several enzymatic reactions that are accessible to nutritional supplementation, and influence renal tubular physiology [52]. Although the metabolic theory stays to be documented, both the influence of the PPAR system on diabetic nephropathy and of C-peptide on PPAR are becoming obvious [53, 54].

In addition, these studies strongly suggest that both renal glomerule and tubule are a major site of C-peptide action. During type 1 diabetes, C-peptide substitution restores in part the functional properties of the renal tubule and, therefore, allows better renal function and metabolic status.

## 4. CONCLUSION

In humans, C-peptide exerts a regulatory and physiologic influence on renal function in patients with type 1 diabetes. Successful islet transplantation has been associated with improvements in kidney graft survival rates and function among uremic patients with type 1 diabetes mellitus and kidney grafts. This suggests that together with the positive effects of normalization of glycometabolic control, successful islet transplantation exerts beneficial effects on kidney function, in part by restoring the C-peptide secretion, a situation closer to the endogenous pancreatic function. Although the mechanisms are not fully understood, a hormonal therapeutic role of C-peptide as an active protective factor for the diabetic kidney should be considered.
